# Analysis of surgery for incurable gastric cancer

**DOI:** 10.1186/s12957-015-0750-z

**Published:** 2015-12-18

**Authors:** Honguang Zhao, Wenhu Chen, Yehua Lin, Jiangfeng Qin, Lifang Wang

**Affiliations:** Department of Thoracic Surgery, Zhejiang Key Laboratory of Diagnosis and Treatment Technology on Thoracic Oncology (Lung and Esophagus), Zhejiang Cancer Hospital, Hangzhou, 310022 People’s Republic of China; Wenzhou Medical University, Wenzhou, 325035 People’s Republic of China; Department of Biochemistry, Institute of Basic Medical Science, Zhejiang Medical College, Hangzhou, 310053 People’s Republic of China; Department of Medical Record, Zhejiang Cancer Hospital, Hangzhou, 310022 People’s Republic of China

**Keywords:** Gastric cancer, Surgery, Incurable, Multiple factor analysis

## Abstract

**Background:**

It is important to evaluate the curability of and avoid unnecessary exploratory surgery for gastric cancer preoperatively. However, no related research has been reported until now. The aim of this study was to evaluate the factors influencing surgery for incurable gastric cancer.

**Methods:**

310 cases of T3–4 gastric cancer patients were analyzed retrospectively, including 141 cases with radical surgery and 169 with surgery for incurable gastric cancer. The incurable factors were categorized as T status (unresectable T4 tumor), N status (unresectable lymph node), peritoneal metastasis, and distant metastasis. *χ*^2^ test and logistic regression were performed to analyze the associations between curability, T status, N status, peritoneal metastasis, or distant metastasis and clinicopathological data.

**Results:**

Esophageal involvement and T grade were associated with curability. Cardia involvement and Borrmann type were associated with T status. Esophageal involvement and T grade were associated with N status. Gastric body involvement, esophageal involvement, and T grade were associated with peritoneal metastasis. Gastric antrum involvement was associated with distant metastasis.

**Conclusions:**

The influencing factors of surgery for incurable gastric cancer should be analyzed preoperatively. Resectability should be evaluated according to these influencing factors combined with imaging analysis.

## Background

Gastric cancer is one of the most frequently diagnosed cancers worldwide. According to GLOBOCAN 2012, 952,000 new cases of gastric cancer were estimated to have occurred in 2012 (6.8 % of the total cancer burden) and gastric cancer ranks fifth among most common malignancies. Approximately 24 % of gastric cancer cases occurred in China [1], and the disease is the second most common cancer in China [2, 3]. Gastric cancer is the third leading cause of cancer-related death globally. Over 95 % of cases have an adenocarcinoma histology, and the disease is often diagnosed at an advanced stage.

The prognosis of gastric cancer has improved with advances in surgical techniques, chemoradiotherapy, and molecular targeted therapy [4]. However, the long-term outcomes of patients with gastric cancer remain poor, particularly for those with advanced disease. The 5-year survival rate in patients with early gastric cancer is 85–100 %, while it is only 5–20 % for advanced gastric cancer patients. Surgery is still the cornerstone of gastric cancer treatment, and locoregional control is important [5]. However, the R0 resection rate for gastric cancer is only 66.7 % with surgery alone [6]. There is no benefit for patients with incurable disease to undergo surgical treatment. Therefore, it is important to evaluate the curability of and avoid unnecessary exploratory surgery for gastric cancer preoperatively. However, no related research has been reported until now.

In our study, the clinicopathological data for gastric cancer for surgery were reviewed. The associations between these clinicopathological factors and curability of gastric cancer were analyzed. Simultaneously, the incurable factors were classified as T status (unresectable T4 tumor), N status (unresectable lymph node), peritoneal metastasis, and distant metastasis. The related clinicopathological factors for each incurable factor were analyzed. This study could provide a reference for the curability evaluation of gastric cancer.

## Methods

### Patients

A total of 1961 cases of primary gastric cancer were treated by surgery at Zhejiang Cancer Hospital (Hangzhou, China) from January 2007 to December 2009. Among them, 1790 cases (91.3 %) received curative resection, which was defined as absence of tumor macroscopically after operation. One hundred seventy-one cases (8.7 %) were incurable (45 cases underwent palliative resection, and 126 cases underwent exploratory or bypass surgery). Two cases were excluded from the 171 incurable cases, because they had other cancers simultaneously. Therefore, 169 incurable cases were analyzed. All of the incurable cases were grades T3–4. Meanwhile, 141 cases with pathological T3–4 stages who underwent radical surgery from January 2007 to August 2007 were chosen as the control group. All cases had undergone both computed tomography (CT) and endoscopy preoperatively. Cases with other cancers simultaneously were excluded from this research.

### Data collection

The clinicopathological data (gender, age, surgery properties, tumor region, esophageal involvement, duodenal involvement, Borrmann type, pathologic type, grading of gastric cancer, and T grade) of all gastric cancer cases with surgery for incurable disease and those with radical surgery were collected. However, not all cases had data for the Borrmann type or grading of gastric cancer. The Borrmann type data were complete in 223 cases, and the grading data were complete in 262 cases.

The tumor staging was determined according to the 2002 American Joint Committee on Cancer (AJCC). The TNM stage system and T grade were applied according to WHO guidelines. Pathological T was used in radical or palliative resection cases. For those cases who had undergone exploratory or bypass surgery, pathological T was impossible; therefore, surgical staging was used. The presence or absence of lymph node metastasis was evaluated according to the tumor node metastasis classification based on the postoperative histopathologic examination. Incurable factors were categorized as T status (unresectable T4 tumor), N status (unresectable lymph node), peritoneal metastasis, and distant metastasis.

### Statistical analysis

Statistical analyses were performed using SPSS 16.0 (SPSS Inc., Chicago, IL, USA). The statistical analyses were as follows:

Step 1: The associations between curability, T status, N status, peritoneal metastasis, or distant metastasis and clinicopathological data were analyzed by chi-square test.

Step 2: Binary logistic regression (backward: conditional method) was performed using curability, T status, N status, peritoneal metastasis, and distant metastasis as dependent variables and the related clinicopathological data from step 1 (except Borrmann type and grading) as covariates, because some cases had no available Borrmann type or grading data.

Step 3: If Borrmann type or grading factor was the related factor, binary logistic regression was then performed again using curability, T status, N status, peritoneal metastasis, or distant metastasis as dependent variables and the related clinicopathological data from step 2 plus Borrmann type or grading as covariates in those cases with complete Borrmann type or grading factor.

Step 4: If Borrmann type or grading factor was not the related factor by binary logistic regression from step 3, the results in step 2 were the final result. However, if Borrmann type or grading factor was the related factor, the results in step 3 were the final result.

## Results

### Patient characteristics

310 cases of gastric cancer were included in our study. Of these cases, 141 underwent radical surgery and 169 cases underwent surgery for incurable disease (44 cases underwent palliative resection, and 125 cases underwent exploratory or bypass surgery). The mean age was 58.49 ± 10.28 years (range 29 to 61 years) in the radical surgery group and 56.75 ± 11.57 years (range 27 to 77 years) in the incurable surgery group (*p* = 0.167). The radical surgery group consisted of 106 male cases (75.2 %) and 35 female cases (24.8 %), and the incurable surgery group consisted of 120 male cases (71.0 %) and 49 female cases (29.0 %) (*p* = 0.411). One or more factors existed in each incurable case. They were categorized as T status in 97 cases, N status in 53 cases, peritoneal metastasis in 103 cases, and distant metastasis in 19 cases (hepatic metastasis in 17 cases, supraclavicular lymph node metastasis in 2 cases). Borrmann type data were complete in 223 cases, and grading data were complete in 262 cases.

### Associations between curability, T status, N status, peritoneal metastasis, or distant metastasis and clinicopathological factors

Gender, age, surgical properties, tumor region, esophageal involvement, duodenal involvement, pathologic type, and T grade were analyzed in 310 cases, Borrmann type was analyzed in 223 cases, and the grading of gastric cancer was analyzed in 262 cases. The results showed that cardia involvement, gastric antrum involvement, esophageal involvement, T grade, Borrmann type, and grading of gastric cancer were associated with curability in univariate analysis by chi-square test (Table 1).

**Table 1:** Associations between curability and clinicopathological data

		Curability (*n* = 310)		
		Yes	No	*p*
Gender	Male	106	120	0.411
	Female	35	49	
Age	<60 years	72	96	0.312
	≥60 years	69	73	
Cardia	Not involved	86	143	0.000^**^
	Involved	55	26	
Gastric body	Not involved	65	75	0.762
	Involved	76	94	
Gastric antrum	Not involved	72	51	0.000^**^
	Involved	69	118	
Number of regions	1	84	106	0.355
	2	55	57	
	3	2	6	
Esophagus	Not involved	97	161	0.000^**^
	Involved	44	8	
Duodenum	No	136	157	0.171
	Yes	5	12	
Signet ring cell carcinoma	No	111	124	0.121
	Partly	26	31	
	Mainly	4	14	
T grade	3	136	72	0.000^**^
	4	5	97	
Borrmann type	I + II	49	33	0.000^**^
	III	86	28	
	IV	6	21	
Grading	Well and moderately differentiated	20	9	0.046^*^
	Poorly differentiated	115	118	

*p < 0.05, **p < 0.01

Cardia involvement, gastric antrum involvement, esophageal involvement, duodenal involvement, T grade, N status, peritoneal metastasis, and Borrmann type were associated with T status. Cardia involvement, esophageal involvement, T grade, and Borrmann type were associated with N status (Table 2). Age, gastric body involvement, the number of regions, esophageal involvement, signet ring cell carcinoma, T grade, Borrmann type, and grading were associated with peritoneal metastasis. Gastric antrum involvement was associated with distant metastasis. The number of regions was nearly related to distant metastasis (Table 3).

**Table 2:** Associations between the T status or N status and clinicopathological data

		T status			N status		
		No	Yes	*p*	No	Yes	*p*
Gender	Male	155	71	0.938	187	39	0.902
	Female	58	26		70	14	
Age	<60 years	115	53	0.915	139	29	0.933
	≥60 years	98	44		118	24	
Cardia	Not involved	142	87	0.000^**^	184	45	0.045
	Involved	71	10		73	8	
Gastric body	Not involved	93	47	0.432	118	22	0.557
	Involved	120	50		139	31	
Gastric antrum	Not involved	99	24	0.000^**^	106	17	0.214
	Involved	114	73		151	36	
Number of regions	1	127	63	0.657	159	31	0.326
	2	80	32		90	22	
	3	6	2		8	0	
Esophagus	Not involved	166	92	0.000^**^	207	51	0.005
	Involved	47	5		50	2	
Duodenum	No	206	87	0.012^*^	243	50	0.951
	Yes	7	10		14	3	
Signet ring cell carcinoma	No	160	75	0.838	193	42	0.787
	Partly	41	16		49	8	
	Mainly	12	6		15	3	
T grade	3	208	0	0.000^**^	187	21	0.000
	4	5	97		70	32	
Borrmann type	I + II	70	12	0.000^**^	76	6	0.002^**^
	III	98	16		108	6	
	IV	12	15		20	7	
Grading	Well and moderately differentiated	24	5	0.275	26	3	0.404
	Poorly differentiated	171	62		195	38	

*p < 0.05, **p < 0.01

**Table 3:** Associations between peritoneal metastasis or distant metastasis and clinicopathological data

		Peritoneal metastasis				Distant metastasis			
		No	Yes	*χ* ^2^	*p*	No	Yes	*χ* ^2^	*p*
Gender	Male	153	73	0.322	0.571	211	15	0.374	0.541
	Female	54	30			80	4		
Age	<60 years	104	64	3.92	0.048	155	13	1.65	0.199
	≥60 years	103	39			136	6		
Cardia	Not involved	148	81	1.818	0.178	214	15	0.27	0.603
	Involved	59	22			77	4		
Gastric body	Not involved	108	32	12.371	0.000^**^	130	10	0.456	0.499
	Involved	99	71			161	9		
Gastric antrum	Not involved	81	42	0.078	0.780	120	3	4.826	0.028^*^
	Involved	126	61			171	16		
Number of regions	1	132	58	7.045	0.030^*^	179	11	5.106	0.078
	2	73	39			106	6		
	3	2	6			6	2		
Esophagus	Not involved	162	96	11.001	0.001^**^	240	18	1.921	0.166
	Involved	45	7			51	1		
Duodenum	No	194	99	0.762	0.383	276	17	0.993	0.319
	Yes	13	4			15	2		
Signet ring cell carcinoma	No	164	71	5.748	0.056	220	15	1.28	0.527
	Partly	35	22			53	4		
	Mainly	8	10			18	0		
T grade	3	153	55	13.111	0.000^**^	198	10	1.918	0.166
	4	54	48			93	9		
Borrmann type	I + II	64	18	16.338	0.000^**^	77	5	1.488	0.475
	III	96	18			111	3		
	IV	13	14			26	1		
Grading	Well and moderately differentiated	25	4	3.822	0.051	28	1	0.497	0.481
	Poorly differentiated	160	73			217	16		

*p < 0.05, **p < 0.01

### Multivariate analyses for curability, T status, N status, peritoneal metastasis, and distant metastasis

Esophageal involvement and T grade were associated with curability in 310 cases in multivariate analysis by logistic regression. Next, the relationships of Borrmann type, esophageal involvement, and T grade with curability were analyzed in 223 cases by logistic regression. The relationship between grading, esophageal involvement, or T grade with incurable factors was analyzed in 223 cases by logistic regression. The results showed that both esophageal involvement and the T grade were associated with curability (Table 4).

**Table 4:** Multivariate analyses for curability

		Curability			
		OR	*p*	aOR	*p*
Esophageal	Not involved	1		1	
	Involved	0.110 (0.005–0.242)	0.000	0.093 (0.032–0.266)	0.000^**^
T grade	3	1		1	
	4	36.644 (14.270–94.103)	0.000	39.957 (14.457–110.432)	0.000^**^

**p < 0.01

Cardia involvement and duodenal involvement were nearly related to the T status in 310 cases in multivariate analysis by logistic regression. Next, the relationships of Borrmann type, cardia involvement, and duodenal involvement with incurable factors were analyzed in 223 cases by logistic regression. The results showed that duodenal involvement was not related to incurable factors in multivariate analysis. However, cardia involvement and Borrmann type were associated with T status (Table 5).

**Table 5:** Multivariate analyses for the T status

		T status			
		OR	*p*	aOR	*p*
Cardia	Not involved	1		1	
	Involved	0.263 (0.099–0.703)	0.008	0.275 (0.101–0.748)	0.011^*^
Borrmann type	I + II	1		1	
	III	0.952 (0.424–2.138)	0.906	0.832 (0.363–1.908)	0.665
	IV	2.700 (1.658–4.397)	0	2.767 (1.647–4.650)	0.000^**^

*p < 0.05, **p < 0.01

Esophageal involvement and T grade were related to N status in 310 cases in multivariate analysis by logistic regression. The relationships of Borrmann type, esophageal involvement, and T grade with N status were analyzed in 223 cases by logistic regression. The results showed that esophageal involvement and T grade were associated with N status (Table 6).

**Table 6:** Multivariate analyses for the N status

		N status			
		OR	*p*	aOR	*p*
Esophageal	Not involved	1		1	
	Involved	0.162 (0.038–0.689)	0.014	0.220 (0.051–0.951)	0.043^*^
T grade	3	1		1	
	4	4.071 (2.201–7.531)	0	3.581 (1.921–6.676)	0.000^**^

*p < 0.05, **p < 0.01

Gastric body involvement, esophageal involvement, and T grade were related to peritoneal metastasis in 310 cases in multivariate analysis by logistic regression. The relationships of Borrmann type, gastric body involvement, esophageal involvement, and T grade with peritoneal metastasis were analyzed in 223 cases by logistic regression. The relationships of grading, gastric body involvement, esophageal involvement, and T grade with peritoneal metastasis were analyzed in 223 cases by logistic regression. The results showed that gastric body involvement, esophageal involvement, and T grade were associated with peritoneal metastasis (Table 7).

**Table 7:** Multivariate analyses for peritoneal metastasis

		Peritoneal metastasis			
		OR	*p*	aOR	*p*
Gastric body	Not involved	1		1	
	Involved	2.420 (1.470–3.985)	0.001	2.576 (1.531–4.334)	0.000^**^
Esophageal	Not involved	1		1	
	Involved	0.263 (0.114–0.605)	0.002	0.307 (0.130–0.728)	0.000^**^
T grade	3	1		1	
	4	2.473 (1.506–4.061)	0	2.348 (1.397–3.946)	0.000^**^

**p < 0.01

Gastric antrum involvement was associated with distant metastasis in 310 cases in multivariate analysis by logistic regression (Table 8).

**Table 8:** Multivariate analyses for distant metastasis

		Distant metastasis	
		OR	*p*
Gastric antrum	Not involved	1	
	Involved	3.743 (1.067–13.129)	0.039^*^

*p < 0.05

## Discussion

Although incurable gastrectomy may lead to a higher quality of life and longer survival time in some reports [7–10], the data are still disputable [11, 12]. Incurable resection is associated with significant perioperative morbidity and mortality as well as a limited overall survival; therefore, it should be performed judiciously [11]. According to the National Comprehensive Cancer Network (NCCN) guidelines version 1.2014, gastric resections should be reserved for the palliation of symptoms (e.g., obstruction or uncontrollable bleeding) in patients with incurable disease. In fact, in most cases, the decision of palliative resection was made when the tumor was found to be incurable by surgery in those patients scheduled for potentially curative resection. Over the last few decades, surgery as the sole form of treatment has been replaced by different forms of multidisciplinary treatment for gastric cancer worldwide. The R0 resection rate has been significantly increased with neoadjuvant chemotherapy for gastric cancer [6].

However, the evaluation of curability for gastric cancer is not completely accurate based on current imaging technology. The current gold standard for T staging is endoscopic ultrasonography (EUS), which has an accuracy between 65 and 92 % [13] and a sensitivity and specificity of 88 and 100 % for T1, 82 and 96 % for T2, 90 and 95 % for T3, and 99 and 97 % for T4, respectively [14]. Multi-detector computed tomography (MDCT) for T staging is less accurate than EUS, although the sensitivity and specificity of serosa involvement are similar to those of EUS [13, 14]. A meta-analysis involving nine studies utilizing positron emission tomography (PET) to evaluate gastric cancer reported that, despite the inability to stage gastric cancer by tumor depth, PET has a pooled primary tumor detection rate of 80 % in identifying the existence of gastric cancer [15]. The sensitivity and specificity for N staging using EUS are approximately 50–60 and 85–95 % [14], respectively, and MDCT is not superior to EUS [13, 14]. PET can evaluate node metabolism using the standardized uptake value (SUV) in addition to acquiring the size of the lymph nodes. However, the mean SUV noted for N staging can also vary, with overall values ranging from 4.5 to 6.8 and an overall accuracy ranging from 17.7 to 79.2 % [15]. The role of PET/CT is limited in T staging of primary tumors due to its low spatial resolution, preventing the evaluation of adjacent organ invasion [16]. For N staging, PET/CT is considered to have similar diagnostic performance to that of contrast-enhanced CT [17]. CT was superior to PET in terms of sensitivity (*p* < 0.0001), and PET was superior to CT in terms of specificity (*p* < 0.0001) and the positive predictive value (PPV) (*p* = 0.05) [18].

For the preoperative diagnosis of peritoneal carcinomatosis (PC) of gastric cancer origin, useful imaging techniques include ultrasonography, CT, magnetic resonance imaging, and 18F-fluorodeoxyglucose PET-CT (FDG PET-CT), but all of these imaging techniques have major limitations in diagnosing PC because of the low-volume density of peritoneal nodules. Concerning PC of gastric cancer origin, Yang et al. [19] reported a PET-CT accuracy of 87 %, with a sensitivity and specificity of 72.7 and 93.6 %, respectively, which were better than those for CT; however, for primary gastric cancer and lymph node metastases, the accuracy for PET-CT is 54 %. CT is not accurate (8–17 % of sensitivity), particularly for malignant granulations less than 5 mm in diameter and small bowel nodulations. Due to the low accuracy provided by the imaging, the main diagnostic methods currently used to evaluate the peritoneal surface are diagnostic laparoscopy or laparotomy and peritoneal cytological examination, which show greater accuracies in diagnosing PC [20].

Therefore, the risk factors of surgery for incurable gastric cancer need to be identified to evaluate curability. However, no related research has been reported until now. To our knowledge, the present study may be the first to evaluate the risk factors for surgery of incurable gastric cancer.

Our study found that esophageal involvement and T grade were associated with curability. Cardia involvement and Borrmann type were associated with T status. Esophageal involvement and T grade were associated with N status. Gastric body involvement, esophageal involvement, and T grade were associated with peritoneal metastasis. Gastric antrum involvement was associated with distant metastasis.

T grade is an important factor in tumor evaluation. According to the AJCC international staging standard, the T grade can be classified as T1 to T4. Patients who present with T4 gastric cancer (~20 % of the patient population) will benefit from aggressive en bloc surgical resection and should not be considered to have unresectable tumors [21]. However, some T4 tumors were unresectable, forming the T status of surgery for incurable gastric cancer. The risk of regional nodal involvement increases with deep penetration through the gastric wall [22], and the nodal extension of the cancer occurs gradually, radiating from the primary location via the lymphatic system [23, 24]. Nodal metastases are observed in 3–5 % of gastric carcinomas limited to the mucosa. Of these nodal metastases, 11–25 % extend to the submucosa, 50 % of the latter reach the muscularis (T2), and 83 % of the latter extend to the serosa (T3) [25, 26]. Our study revealed that T grade is correlated with the N status and peritoneal dissemination with surgery for incurable disease.

Esophageal involvement is a protective factor for surgery for incurable disease and N status and peritoneal dissemination with surgery for incurable disease. Cardia involvement is a protective T factor for surgery for incurable disease likely because esophageal or cardia involvement can cause digestive tract obstruction and can be diagnosed earlier. Moreover, even if the tissues at the gastroesophageal junction are invaded, they can be resected easily.

The Borrmann type is divided into four types, and they infiltrate in different ways. We discovered that the Borrmann type is related to the T status for surgery for incurable disease.

The penetration and dissemination of gastric cancer cells into the peritoneal cavity is referred to as PC [27], which is considered stage IV disease. Peritoneal dissemination occurs more frequently than do hematogenous metastases. The prognosis of gastric cancer patients with PC is poor. Currently, in the intraoperative abdominal examination, peritoneal seeding is found in 10–20 % of patients scheduled for potentially curative resection and in 40 % of those at stage II–III [28–30]. Cell distribution into the peritoneal cavity is also dependent on physical factors: the tumor primary site, effects of gravity, the presence of fluids (e.g., ascites and mucus), and intrinsic biological aggressiveness [31].

We discovered that gastric body involvement and T grade were risk factors for peritoneal dissemination. Peritoneal dissemination occurs only when the cancer invades into the serosa, particularly when the gastric body is involved. Esophageal involvement is a protective factor for peritoneal dissemination with surgery for incurable disease. Esophageal involvement can cause digestive tract obstruction and be diagnosed earlier. Cancer more readily invades the tissue around the gastroesophageal junction than the peritoneal cavity, even if the serosa has been invaded.

Our study revealed that gastric antrum involvement is a risk factor for distant metastasis. The blood from the gastric antrum flows to the portal vein through the right gastric vein, possibly leading to liver metastasis.

We believe that the factors influencing surgery for incurable disease should be analyzed preoperatively. Resectability should be evaluated according to these influencing factors combined with imaging analysis. Laparoscopic exploration is feasible when gastric cancer is potentially incurable by surgery.

## Conclusions

Esophageal involvement and the T grade were associated with curability. Cardia involvement and Borrmann type were associated with the T status. Esophageal involvement and the T grade were associated with the N status. Gastric body involvement, esophageal involvement, and the T grade were associated with peritoneal metastasis. Gastric antrum involvement was associated with distant metastasis. The factors influencing surgery for incurable disease should be analyzed preoperatively. Resectability should be evaluated according to these influencing factors combined with imaging analysis.

### Consent to publish

We have obtained consent to publish from the participant to report individual patient data with reference number 2010–002 from the Ethics Committee of Zhejiang Medical College.

## References

[1] Lin Y, Ueda J, Kikuchi S, Totsuka Y, Wei WQ, Qiao YL (2011). Comparative epidemiology of gastric cancer between Japan and China. World J Gastroenterol.

[2] Siegel R, Naishadham D, Jemal A (2013). Cancer statistics, 2013. CA Cancer J Clin.

[3] Chen W, Zheng R, Zhang S, Zhao P, Li G, Wu L (2013). Report of incidence and mortality in China cancer registries, 2009. Chin J Cancer Res.

[4] Hartgrink HH, Jansen EP, van Grieken NC, van de Velde CJ (2009). Gastric cancer. Lancet.

[5] Songun I, Putter H, Kranenbarg EM, Sasako M, van de Velde CJ (2010). Surgical treatment of gastric cancer: 15-year follow-up results of the randomised nationwide Dutch D1D2 trial. Lancet Oncol.

[6] Miao RL, Wu AW (2014). Towards personalized perioperative treatment for advanced gastric cancer. World J Gastroenterol.

[7] Haugstvedt T, Viste A, Eide GE, Söreide O (1989). The survival benefit of resection in patients with advanced stomach cancer: the Norwegian multicenter experience. Norwegian Stomach Cancer Trial. World J Surg.

[8] Hartgrink HH, Putter H, Klein Kranenbarg E, Bonenkamp JJ, van de Velde CJ, Dutch Gastric Cancer Group (2002). Value of palliative resection in gastric cancer. Br J Surg.

[9] Samarasam I, Chandran BS, Sitaram V, Perakath B, Nair A, Mathew G (2006). Palliative gastrectomy in advanced gastric cancer: is it worthwhile?. ANZ J Surg.

[10] Sun J, Song Y, Wang Z, Chen X, Gao P, Xu Y (2013). Clinical significance of palliative gastrectomy on the survival of patients with incurable advanced gastric cancer: a systematic review and meta-analysis. MC Cancer..

[11] Schmidt B, Look-Hong N, Maduekwe UN, Chang K, Hong TS, Kwak EL (2013). Noncurative gastrectomy for gastric adenocarcinoma should only be performed in highly selected patients. Ann Surg Oncol.

[12] Ouchi K, Sugawara T, Ono H, Fujiya T, Kamiyama Y, Kakugawa Y (1998). Therapeutic significance of palliative operations for gastric cancer for survival and quality of life. J Surg Oncol.

[13] Kwee RM, Kwee TC (2007). Imaging in local staging of gastric cancer: a systematic review. J Clin Oncol.

[14] Puli SR, Batapati Krishna Reddy J, Bechtold ML, Antillon MR, Ibdah JA (2008). How good is endoscopic ultrasound for TNM staging of gastric cancers? A meta-analysis and systematic review. World J Gastroenterol.

[15] Seevaratnam R, Cardoso R, McGregor C, Lourenco L, Mahar A, Sutradhar R (2012). How useful is preoperative imaging for tumor, node, metastasis (TNM) staging of gastric cancer? A meta-analysis. Gastric Cancer.

[16] Yun M (2014). Imaging of gastric cancer metabolism using 18F-FDG PET/CT. J Gastric Cancer.

[17] Kim EY, Lee WJ, Choi D, Lee SJ, Choi JY, Kim BT (2011). The value of PET/CT for preoperative staging of advanced gastric cancer: comparison with contrast-enhanced CT. Eur J Radiol.

[18] Kim SK, Kang KW, Lee JS, Kim HK, Chang HJ, Choi JY (2006). Assessment of lymph node metastases using 18F-FDG PET in patients with advanced gastric cancer. Eur J Nucl Med Mol Imaging.

[19] Yang QM, Kawamura T, Itoh H, Bando E, Nemoto M, Akamoto S (2008). Is PET-CT suitable for predicting lymph node status for gastric cancer?. Hepatogastroenterology.

[20] Yonemura Y, Elnemr A, Endou Y, Hirano M, Mizumoto A, Takao N (2010). Multidisciplinary therapy for treatment of patients with peritoneal carcinomatosis from gastric cancer. World J Gastrointest Oncol.

[21] Shchepotin IB, Chorny VA, Nauta RJ, Shabahang M, Buras RR, Evans SR (1998). Extended surgical resection in T4 gastric cancer. Am J Surg.

[22] Lawson JD, Sicklick JK, Fanta PT (2011). Gastric cancer. Curr Probl Cancer.

[23] Peeters KC, Hundahl SA, Kranenbarg EK, Hartgrink H, van de Velde CJ (2005). Low Maruyama index surgery for gastric cancer: blinded reanalysis of the Dutch D1-D2 trial. World J Surg.

[24] McCulloch P, Nita ME, Kazi H, Gama-Rodrigues JJ (2012). WITHDRAWN: extended versus limited lymph nodes dissection technique for adenocarcinoma of the stomach. Cochrane Database Syst Rev.

[25] Oñate-Ocaña LF, Aiello-Crocifoglio V, Mondragón-Sánchez R, Ruiz-Molina JM (2000). Survival benefit of D2 lympadenectomy in patients with gastric adenocarcinoma. Ann Surg Oncol.

[26] de Gara CJ, Hanson J, Hamilton S (2003). A population-based study of tumor-node relationship, resection margins, and surgeon volume on gastric cancer survival. Am J Surg.

[27] Gill RS, Al-Adra DP, Nagendran J, Campbell S, Shi X, Haase E (2011). Treatment of gastric cancer with peritoneal carcinomatosis by cytoreductive surgery and HIPEC: a systematic review of survival, mortality, and morbidity. J Surg Oncol.

[28] Kodera Y, Yamamura Y, Shimizu Y, Torii A, Hirai T, Yasui K (1999). Peritoneal washing cytology: prognostic value of positive findings in patients with gastric carcinoma undergoing a potentially curative resection. J Surg Oncol.

[29] Roviello F, Caruso S, Marrelli D, Pedrazzani C, Neri A, De Stefano A (2011). Treatment of peritoneal carcinomatosis with cytoreductive surgery and hyperthermic intraperitoneal chemotherapy: state of the art and future developments. Surg Oncol.

[30] Sugarbaker PH (1998). Intraperitoneal chemotherapy and cytoreductive surgery for the prevention and treatment of peritoneal carcinomatosis and sarcomatosis. Semin Surg Oncol.

[31] Montori G, Coccolini F, Ceresoli M, Catena F, Colaianni N, Poletti E (2014). The treatment of peritoneal carcinomatosis in advanced gastric cancer: state of the art. Int J Surg Oncol.

